# Adjusting a cancer mortality-prediction model for disease status-related eligibility criteria

**DOI:** 10.1186/1471-2288-11-64

**Published:** 2011-05-11

**Authors:** Millennia Foy, Xing Chen, Marek Kimmel, Olga Y Gorlova

**Affiliations:** 1 Brown Foundation Institute of Molecular Medicine, University of Texas Health Science Center at Houston, 1825 Pressler St, Houston, TX 77030, USA; 2 Department of Epidemiology, University of Texas MD Anderson Cancer Center, 1515 Holcombe Blvd, Houston, TX 77030, USA; 3 Department of Statistics, Rice University, 6100 Main St, Houston, TX 77005, USA

## Abstract

**Background:**

Volunteering participants in disease studies tend to be healthier than the general population partially due to specific enrollment criteria. Using modeling to accurately predict outcomes of cohort studies enrolling volunteers requires adjusting for the bias introduced in this way. Here we propose a new method to account for the effect of a specific form of healthy volunteer bias resulting from imposing disease status-related eligibility criteria, on disease-specific mortality, by explicitly modeling the length of the time interval between the moment when the subject becomes ineligible for the study, and the outcome.

**Methods:**

Using survival time data from 1190 newly diagnosed lung cancer patients at MD Anderson Cancer Center, we model the time from clinical lung cancer diagnosis to death using an exponential distribution to approximate the length of this interval for a study where lung cancer death serves as the outcome. Incorporating this interval into our previously developed lung cancer risk model, we adjust for the effect of disease status-related eligibility criteria in predicting the number of lung cancer deaths in the control arm of CARET. The effect of the adjustment using the MD Anderson-derived approximation is compared to that based on SEER data.

**Results:**

Using the adjustment developed in conjunction with our existing lung cancer model, we are able to accurately predict the number of lung cancer deaths observed in the control arm of CARET.

**Conclusions:**

The resulting adjustment was accurate in predicting the lower rates of disease observed in the early years while still maintaining reasonable prediction ability in the later years of the trial. This method could be used to adjust for, or predict the duration and relative effect of any possible biases related to disease-specific eligibility criteria in modeling studies of volunteer-based cohorts.

## Background

Prediction models are a valuable tool for both personalized risk prediction based on risk factors, and for analyzing a population of subjects who are screened for a disease or undergo another preventative intervention, whereby the comparison of observed versus predicted (in the absence of intervention) number of outcomes, such as mortality, can demonstrate whether the intervention has a desired effect. However, a problem for the generalization of results from prediction models is posed by the so-called healthy volunteer effect. Healthy volunteer effect or healthy volunteer bias are terms used to describe the well-documented [1-8] observation of lower rates of disease observed in cohorts enrolling volunteers as compared to the general population. This effect can be seen when volunteers are healthier than average for the general population, which is caused in part by the eligibility requirements of the study. We are studying this latter effect, which is particularly noticeable in the early years of a study. When a prediction model uses data from a cohort of volunteers and then is applied to the general population, the model is likely to under-predict the number of endpoints; conversely, in a situation when the model is fitted to data from the general population and needs to be validated in a cohort, the model may over-predict the number of endpoints in the early years of follow-up. The bias is likely to be stronger if the cohort was specifically organized to study the disease in question and the eligibility criteria explicitly excluded subjects already diagnosed with the disease or having symptoms indicative of the disease. For example, in the context of lung cancer (LC) mortality (used as the outcome in the present paper), the bias is expected to be stronger in the Carotene and Retinol Efficacy Trial (CARET), a lung cancer chemoprevention trial (used here for validation), as compared to Cancer Prevention Study I (CPS-I) or Nurses' Health Study (NHS) (used for model fitting) that investigate multiple health outcomes and do not focus specifically on lung cancer.

In this paper, we propose a method of adjusting for biases related to disease-specific eligibility criteria (briefly, *eligibility-related bias*) using a simple modeling approach. In order to adjust for the eligibility-related bias, we assume that for each individual there is a time interval prior to LC death in which an individual would not be eligible or likely to volunteer for a study. For our study, this interval is modeled as the time between clinical diagnosis and death from LC. However, this time interval may also be extended to include the presence of symptoms prior to clinical diagnosis. Using survival time data from newly diagnosed lung cancer patients treated at MD Anderson Cancer Center, we estimate the distribution of this time interval for lung cancer. Further, we use an exponential distribution to approximate the empirical distribution of this interval.

**Figure 1:**
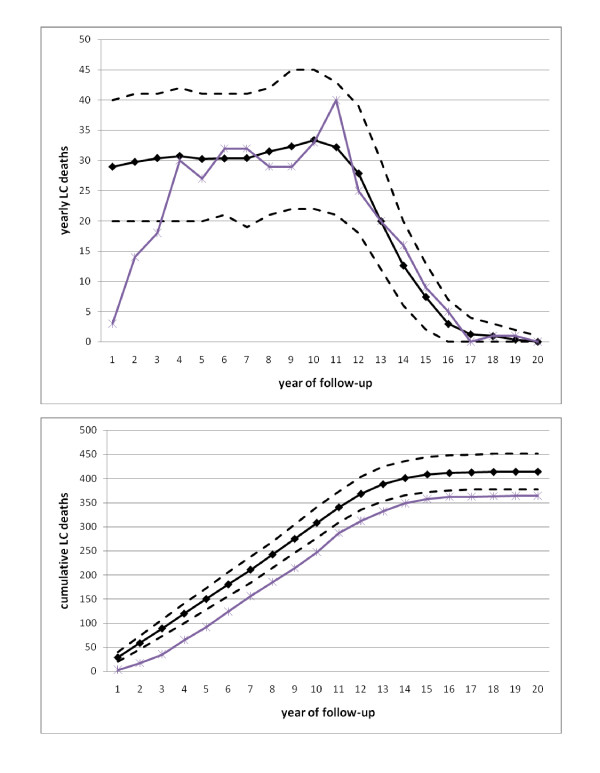
**Observed and predicted lung cancer deaths in CARET**. Black solid line denotes prediction while dashed lines are confidence limits. Purple line denotes observed LC deaths. Top: Annual; Bottom: Cumulative.

The method is then used to adjust predictions of lung cancer mortality in the control arm of the Carotene and Retinol Efficacy Trial (CARET) [9]. A model previously developed [10] is able to accurately predict lung cancer mortality in the CARET cohort for the later years of follow-up (4-20) but overestimates mortality in the early years (1-3) as seen in Figure 1 causing the cumulative LC deaths to be overestimated as a result of the higher predictions made for the first 3 years. There is some indication that the eligibility-related bias in CARET is present during the first 3 years of follow-up as seen in the observed LC deaths (Figure 1). Although person-years are steady in years 1-3 (additional file 1: Figure S1), observed LC mortality is increasing rapidly over this interval. Even though observed mortality can behave somewhat randomly, the magnitude of increase along with the large sample size of the cohort (N = 6877) can be suggestive of the eligibility-related bias. Using the proposed adjustment, we are able to accurately predict the number of lung cancer deaths in CARET over the entire length of follow-up.

## Methods

In order to estimate the distribution of the time interval between clinical lung cancer diagnosis and death from lung cancer, we examined data on lung cancer survival times from 1,190 newly diagnosed LC patients at the University of Texas MD Anderson Cancer Center (MDA). Ethical approval was obtained for this study from the MD Anderson IRB. For each individual, data on survival time from LC diagnosis, vital status, and stage of disease at presentation were available. Median survival times were also obtained from SEER-17 for comparison purposes, since SEER is much more likely to be representative of the general population. The MDA estimates, on the other hand, represent the longer end of the survival distribution, which in modeling would result in fewer deaths and thus could be interpreted as a conservative comparison with a group undergoing any preventative intervention.

Using the data from MDA, Kaplan-Meier (KM) survival curves were generated stratified by AJCC lung cancer stage (I-IV). The individual survival curves by stage were then re-weighted using the stage distribution observed in SEER-17 [11] for lung cancers diagnosed during the year 2000 (I: 16.22%, II: 2.91%, III: 32.64%, IV: 48.23%), to obtain an overall lung cancer survival curve. An exponential distribution was then used to approximate the overall lung cancer survival curve in order to simplify the adjustment. The parameter of this distribution was found by using the KM estimate of the median survival time in the overall KM curve and converting it to the mean λ of the exponential distribution, using the following formula *λ*=[1/ln(2)]*x_med_*.

This exponential distribution is then used in conjunction with the mortality model, as an adjustment for the eligibility-related bias, in order to simulate LC mortality in the control arm of CARET for comparison to the observed mortality.

### Lung Cancer Mortality Model

Predictions and simulations of lung cancer mortality are carried out using a two-stage clonal expansion (TSCE) model [12] modified and validated by Foy et al 2010 [10]. Details about the TSCE model along with parameter estimates can be found in additional file 1 online.

### Carotene and Retinol Efficacy Trial (CARET)

For model validation purposes, data were obtained on the placebo-control arm of the heavy-smokers cohort of the Carotene and Retinol Efficacy Trial (CARET) [9] including data on 6,877 individuals (3797 males and 3080 females) (Table 1). Average observed follow-up was 11.5 years and the longest follow-up was 19.5 years. Data on age at enrollment to CARET, age at end of follow-up, gender, and complete smoking history (age at initiation, age at cessation, and cigarettes smoked per day) were obtained for each individual. Also, data on vital status and cause of death were obtained for calculating observed lung cancer-specific deaths. The observed number of deaths was compared to the number of deaths simulated according to the TSCE model with the adjustment for the eligibility-related bias.

**Table 1:** Characteristics of 3797 males and 3080 females enrolled in the placebo control arm of CARET

Males		Mean	SD
	Age	58.4	5.5
	Pack-years	53	21.5
	Follow-up	11.2	3.4

Females		Mean	SD
	Age	58.2	5.4
	Pack-years	44.4	18.0
	Follow-up	11.8	3.1

Eligibility requirements for the heavy-smokers cohort of CARET were that subjects had at least 20 pack-years of smoking history, were aged 50-69 at enrollment, and were current smokers or had quit within the previous 6 years. Pre-menopausal women, those with a history of cirrhosis or hepatitis within the 12 months prior to enrollment, and those with a history of cancer within 5 years of enrollment were not eligible for CARET. Individuals who had taken less than 50% of the study vitamins during the enrollment period between the first and second visits were also excluded. Since the criteria for this study include disease conditions, and further good compliance to the vitamin protocol, we expect considerable healthy volunteer effect in this study including but probably not limited to the type of bias we are studying. However, we expect that the eligibility-related effect we are studying is likely to dominate as the history of cirrhosis and any cancer, and compliance with the vitamin protocol are not recognized specific risk factors for LC, and because of the high lethality of LC.

Importantly, since lung cancer was not the primary outcome for either CPS-I or NHS (used in fitting the TSCE model), the lung cancer-specific eligibility-related bias is likely to be absent to modest; the lung cancer case-control study, also used for fitting, does not contribute any additional bias. Therefore, the model developed by us in Foy et al. [10] is likely to be accurate for predictions in the general population but may over-predict lung cancer incidence and mortality in a cohort organized to study newly arising lung cancer, such as CARET. Thus, to validate the model using CARET data, the eligibility-related bias has to be taken into account.

### Simulation of Lung Cancer Mortality

Under the TSCE model, let *S*(*t*) denote the probability that an individual will not die of lung cancer by age *t*. For each individual in CARET, *S*(*t*) depends on the individual's complete smoking history, *d *up to age *t *(age at initiation of smoking, age at cessation of smoking, and number of cigarettes smoked per day) and gender, and will be referred to as *S*(*t*;*d*) where *t *= 0 is birth. Since *S*(*t*;*d*) is dependent upon the individual's smoking history up until age *t*, *S*(*t*;*d*) is equivalent for never smokers and smokers prior to initiation. Along with smoking history and gender, the probability of an individual dying from lung cancer during follow-up depends on the age at enrollment, *t*_0_, and age at the end of follow-up, *t*_1_. The following routine was used to simulate lung cancer deaths for each individual from CARET, based on their age and smoking history. Although the following routine is intuitive, it is not exactly representative of the joint distribution. In additional file 1, we show that the underlying distribution does not differ enough to make a difference in the case of this simulation.

For each individual a uniform(0, *S*(*t*_0_; *d*)) random variable, *u*, was drawn.

A. If *u *≤ S(*t*_1_) then no lung cancer death occurs during follow-up and the simulation is retired.

B. If *u *>*S*(*t*_1_) then lung cancer death occurs during follow-up at age, t*, computed by inverting the tail function of the age at death distribution, *u *= *S*(t*). In order to adjust for the eligibility-related bias, the length of time between lung cancer diagnosis and death is simulated (exponentially distributed with a mean of 2 years) and an age at LC diagnosis is calculated by subtracting from the age at death.

a. If the age at LC diagnosis is greater than the age at enrollment the simulation is retired.

b. If the age at LC diagnosis is less than the age at enrollment the simulation is rejected and the individual is simulated again.

According to this routine, for a simulated individual to die from lung cancer they must be diagnosed after the age of enrollment and then die from the disease before the age at the end of follow-up. If they were simulated to die from lung cancer during follow-up but would have been diagnosed previous to their age at enrollment, then their simulation is repeated. The routine is preformed for every individual from CARET to simulate a single trial, and 5000 CARET trials were generated to produce expected cumulative and annual number of lung cancer deaths per follow-up year and corresponding confidence intervals. The expected number of lung cancer deaths is calculated as the mean of the simulated trials and the confidence intervals are estimated using the 2.5% and 97.5% percentiles.

## Results

Figures depicting the KM survival curves by stage and the overall KM curve, calculated as the weighted average of the individual stage curves, are contained in the additional material for both the SEER-17 (diagnosed in the year 2000) and the MDA data. The resulting KM estimate of the median survival time is 17 months between lung cancer diagnosis and death in the MDA patients. Details of the calculations of the estimated interval between diagnosis and death, a comparison of the exponential approximation and the overall KM survival curve, and stage distribution of newly diagnosed cases in SEER-17 for the year 2000 are also included in additional file 1. SEER median survival time for LC-specific mortality is reported as 11 months [11] for cases diagnosed in 2000. The corresponding exponential distribution means are therefore 2.0 years (MDA) and 1.3 years (SEER), and both are used to predict LC deaths in CARET.

The adjustment for the eligibility-related bias does not take into account the fact that the time from LC symptoms to LC death is a random variable, which is not independent of the random time from birth to LC death. In additional file 1, we derive a more rigorous adjustment but then we show that it does not result in further modification of the number of LC deaths.

**Figure 2:**
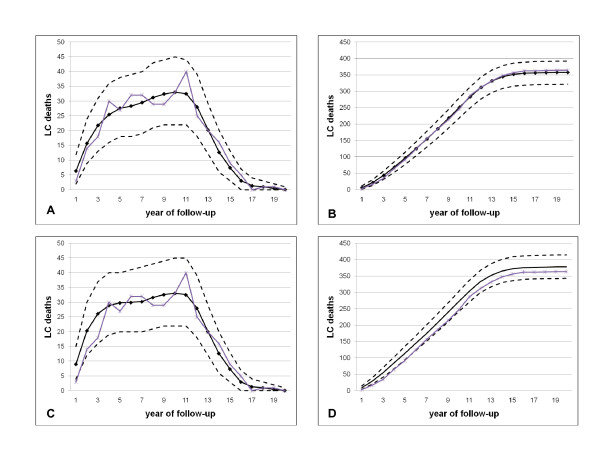
**Observed and predicted lung cancer deaths in CARET using the disease status-related eligibility criteria adjustments**. Black solid line denotes model prediction with dashed lines being confidence limits, and purple line denotes observed LC deaths. Simulations used MDA data (adjustment 2.0 years) (A, yearly; B, cumulative) and SEER data (adjustment 1.3 years) (C, yearly; D, cumulative).

As described in the Methods section, the adjustment and the underlying TSCE model of lung cancer risk were used to simulate LC mortality in CARET. Figure 2 shows the annual and cumulative expected and observed lung cancer mortality using the two adjustment intervals of 1.3 and 2.0 years, reflecting the MDA and SEER estimated intervals. As Table 2 shows, there were 364 lung cancer deaths observed in CARET over the complete follow-up, while the model predicts 357.9 (95% CI: 322, 392) using the MDA adjustment and 377.9 (95% CI: 343, 415) using the SEER based adjustment. However, when no adjustment is applied, the model substantially over-predicts the mortality observed in CARET, with 413.6 predicted deaths (95% CI: 377, 451).

**Table 2:** Predicted and observed number of lung cancer deaths in CARET

	Overall	Males	Females
Observed	364	225	139
No Adjustment	413.6(377,451)	267.3(237,297)	146.6(124,170)
Adjusted			
MDA (2.0 years)	357.9(322,392)	229.3(202,257)	128.3(108,150)
SEER (1.3 years)	377.9(343,415)	242.8(214,272)	135.2(113,158)

## Discussion

The proposed adjustment is able to remove the eligibility-related bias. The interval between diagnosis and death in this study was modeled using an exponential distribution estimated from lung cancer survival time data from both SEER and newly diagnosed patients at MD Anderson Cancer Center (MDA). The median survival time of lung cancer in the patients at MD Anderson is equal to 17 months compared to the SEER reported median survival of 11 months. Although SEER provides good estimates of survival times seen in the general population it is unknown whether these would reflect survival times in a volunteer based cohort which is influenced by the eligibility-related bias especially since volunteers tend to be healthier than the general population [1-8]. A more precise estimate of this interval could come from the lung cancer survival times observed in CARET directly, but this data is lacking for the present study.

A previously suggested approach to deal with healthy volunteer bias was to remove the first few years of follow-up from both the predicted and observed arms [13]. The number of years to remove was chosen arbitrarily and the approach was likely to be too conservative.

In the present paper survival data was used to estimate the interval between diagnosis and death. We may have underestimated this interval due to generally more health conscious behavior seen in volunteers and the health-related exclusion criteria, for example the exclusion of those with a history of cirrhosis or hepatitis within 12 months prior to enrollment in the CARET cohort. However, we expect only a small impact of these criteria on LC mortality prediction since the mentioned conditions are not recognized LC risk factors and because LC is such a fatal disease.

Adjusting for incidence-based mortality in a prediction study may also be thought of and carried out as a deconvolution problem. If the mortality rate and survival time distribution are presumed known, it is possible to use de-convolution to estimate the incidence function. Using the incidence function and survival time distribution it is then possible to remove incidence cases that would have been diagnosed prior to enrollment as described in Pinsky 2009[14]. The method proposed here provides for a simple alternative and has the added bonus of being able to manipulate the length of the interval for example by including the presence of symptoms prior to diagnosis.

One limitation of this study is we did not explore a possibility that the interval between diagnosis and death may differ by gender and/or age at diagnosis. Survival data would indicate that women would have a longer survival than men; however, for the interval between diagnosis and death, the opposite seems true when comparing observed deaths to person-years in each follow-up year. As seen in additional file 1, observed LC deaths are increasing for the first 4 years for men before leveling off and falling while for women the duration of the increase is shorter, even as the observed person-years are slowly decreasing over time. Studies concerning survival by age at diagnosis are conflicting. Palma et al. report no significant differences in survival for older vs. younger patients for stage I non-small cell lung cancer patients [15], while Chang et al. report that older patients (over the age of 67) with stage IA tend to do worse[16]. Yet another study by Bryant and Cerfolio [17] report that patients under the age of 45 with Stage I have a worse prognosis than older patients but not for other stages. Given this information, we chose not to include age in the model for the interval between diagnosis and death. However, for other conditions gender and/or age may be important indicators of survival and need to be included in the adjustment.

The method we propose has another limitation related to the fact that it has been developed for the needs of a specific application. However, the deconvolution method described in additional file 1 is more general in the sense that it applies to non-exponential distributions of the interval from diagnosis to death, given the assumption of independence between age at diagnosis and survival time. Even the latter can be relaxed, although it would lead to computational difficulties. In addition, the eligibility criterion is not necessarily absence of diagnosis; it might be non-occurrence of any specified disease-related event, such as appearance of symptoms.

This method seeks to adjust for the biases caused by specific enrollment criteria. Another bias associated with healthy volunteer bias is self-selection bias, where health conscious individuals are more likely to volunteer for studies. These individuals tend to be healthier than the general population and thus have lower mortality rates. Pinsky et al. [18] have demonstrated a substantially lower than expected overall mortality in both arms of the PLCO screening trial, which was only partially explained by the demographic and risk profile differences between the trial participants and the general population. The authors hypothesized that subjects with certain chronic diseases or conditions that strongly predispose to death over the next 5-10 years were unlikely to volunteer for the PLCO. Therefore, the PLCO trial population does not represent the general population in terms of mortality. Since the self-selection bias is much more difficult to quantify, the method proposed here cannot adjust for it, which is a limitation of this study. However, Pinsky et al [18] observed that the self-selection bias was not as influential in cancer-related mortality as it was in the overall mortality, suggesting that our method may be sufficient to remove most of the healthy volunteer effect on lung cancer mortality.

## Conclusions

In this paper we introduced a new method of adjusting for the eligibility-related bias when predicting the outcome of a cohort study with specific eligibility criteria. The main assumption of this method is that there is a time interval preceding the outcome being studied, in this case death from lung cancer, when individuals would be too sick and/or ineligible to enroll in a study. For this method, we propose using an exponential distribution to approximate the empirical distribution of the time interval between ineligibility for the study and the outcome of interest. In this study we approximated the length of interval as the time between clinical lung cancer diagnosis and death. The mean length of this interval was determined using survival time data from 1190 newly diagnosed lung cancer patients at MD Anderson. Incorporating the resulting exponential distribution into our existing lung cancer model, we were able to more accurately predict the number of lung cancer deaths observed in CARET over the entire length of follow-up, and therefore validate our prediction model. This method could be applied in other modeling efforts in the prediction of outcomes for cohorts enrolling volunteers.

## Competing interests

The authors declare that they have no competing interests.

## Authors' contributions

MF analyzed the survival data, simulated trial results, and drafted the manuscript. XC extracted and advised on the MDA survival data. MK and OYG advised the study and participated in the drafting of the manuscript. MK developed the acceptance probability expression in the Additional file. All authors read and approved the final manuscript.

## Pre-publication history

The pre-publication history for this paper can be accessed here:

http://www.biomedcentral.com/1471-2288/11/64/prepub

## Supplementary Material

Additional file 1 **Supplementary tables and figures**. Click here for file
