# Supplementary material for: Adjusting a cancer mortality-prediction model for disease status-related eligibility criteria
Source: BMC Med Res Methodol. 2011 May 11;11:64. doi: 10.1186/1471-2288-11-64 (PMC3112196; doi:10.1186/1471-2288-11-64)
Supplement: Additional file 1 — Supplementary tables and figures. [file 1471-2288-11-64-S1.PDF]

Adjusting the cancer mortality-prediction model for disease status-related eligibility criteria

Millennia Foy, Xing Chen, Marek Kimmel, Olga Y. Gorlova

Supplement

CARET Study

Figure S1 Person-years of follow-up as recorded in the CARET Study

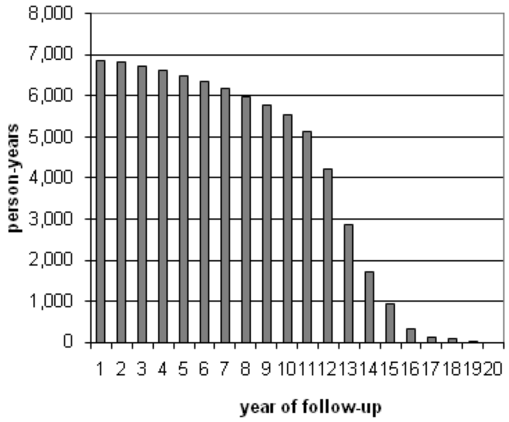

Lung Cancer Mortality Model Predictions and simulations of lung cancer mortality are carried out using a two-stage clonal expansion (TSCE) model [1] modified and validated by Foy et al 2010 [2]. The TSCE model is depicted in Figure S2.

Figure S2 Two-stage Clonal Expansion Model. NC: normal cells; IC: intermediate cells; MC: malignant cells.

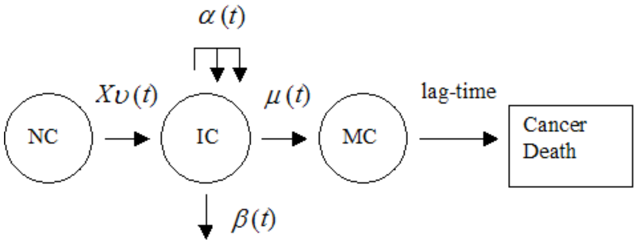

The TSCE model assumes that a normal cell (NC) mutates into an initiated cell (IC) in the first transition, according to a Poisson process with intensity $v(t)$, where t denotes the age. There are X normal cells in the tissue at birth or maturity, depending on the tissue. Then the initiated cell can duplicate or die according to a birth-death process with parameters $\alpha(t)$ and $\beta(t)$ or further mutate into a malignant cell (MC) for the second transition according to a Poisson process with parameter $\mu(t)$. After a lag time, this malignant cell is assumed to develop into a cancerous tumor with probability one. Under piece-wise constant parameters, the cumulative distribution function defining the probability that lung cancer death occurs before time t, can be calculated exactly using recursive formulas outlined by Heidenreich [3].

The TSCE model was fitted using a resampling-based method allowing for estimation of risk factor dependent parameters from the combination of case-control data and prospective mortality rate data [2]. The data on smoking were obtained from the MD Anderson case-control study of lung cancer [4] and the lung cancer incidence/mortality rates were obtained from CPS-I [5] and the Nurses' Health Study [6], for males and females respectively. The following parameters define the TSCE model depending on smoking measured in packs per day (*ppd*) and age t under a fixed lag-time of 6 years [2]. Estimates of the relevant parameters are collected in Table 1.

$$
\begin{aligned}
X &= 10^7 \\
\nu(t) &= \nu_0 X(1 + a_1 \times \sqrt{ppd}) \\
\mu(t) &= \nu_0(1 + a_1 \times \sqrt{ppd}) \\
\alpha(t) &= \alpha_0(1 + a_2 \times \sqrt{ppd}) \\
\gamma(t) &= \alpha(t) - \beta(t) - \mu(t) = \gamma_0(1 + a_2 \times \sqrt{ppd})
\end{aligned}
$$

Table S1 Parameter estimates of the TSCE model

Parameter	α_0	γ_0	$\nu_0 X$	a_1	a_2
Males (CPS-I)	2.99	0.069	2.17	2.66	0.35
Females (NHS)	4.6	0.071	1.93	2.30	0.35

Eligibility requirements for CPS-I were limited to participants being over the age of 30 at and having at least one family member over the age of 45 at the time of enrollment. Nurses' Health Study recruited married registered nurses between the ages of 30 and 55. Since there were no eligibility requirements related to being disease free at the time of enrollment in these studies, we assume that any healthy volunteer effect is probably small in magnitude. Further, the model was only partially informed by tabled mortality rate data from these studies which both included more than 12 years of follow-up. Therefore, we assume that the model was not significantly impacted by any healthy volunteer effect that may have been present in these studies.

Kaplan-Meier (KM) Survival Curves and Interval Estimation Figure S3 shows the KM survival curves by stage and the overall KM curve calculated as the weighted average of the individual stage curves. The weights came from the observed lung cancer incidence proportions reported in SEER 17 (Table S2).

Figure S3 Lung cancer survival curves by stage. Top: MD Anderson LC patients; Bottom: SEER.

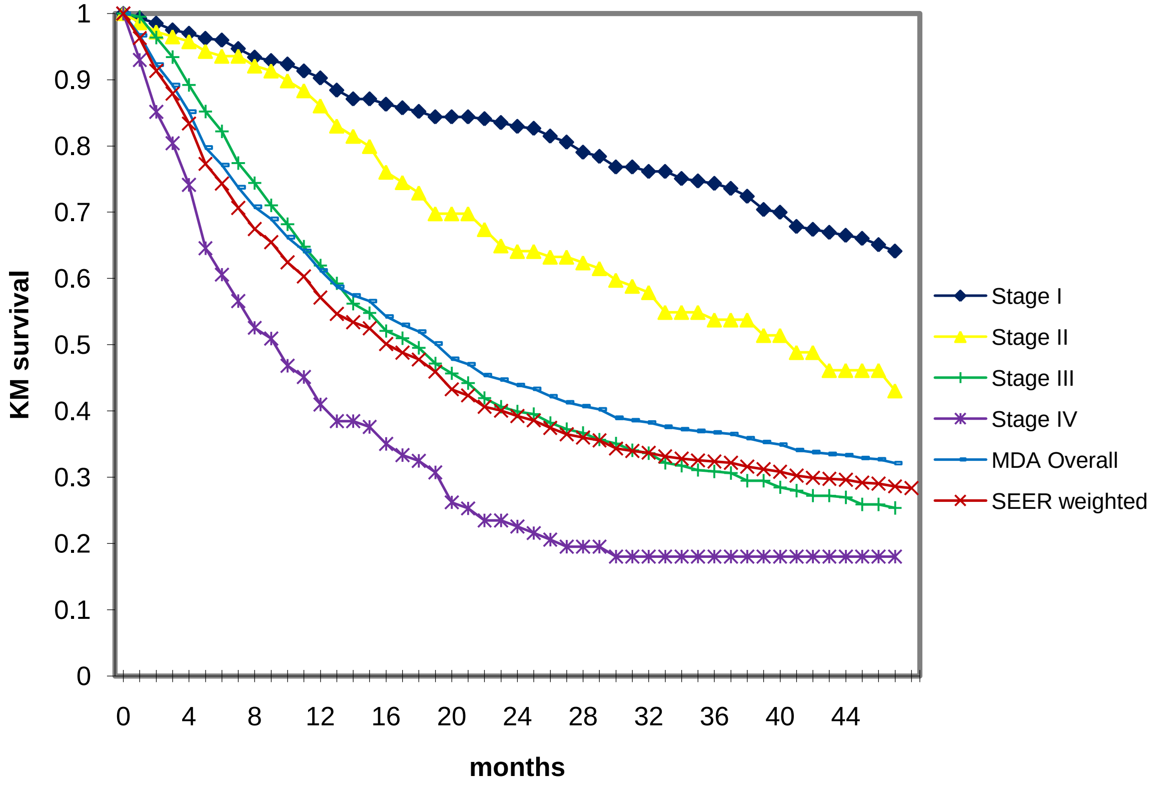

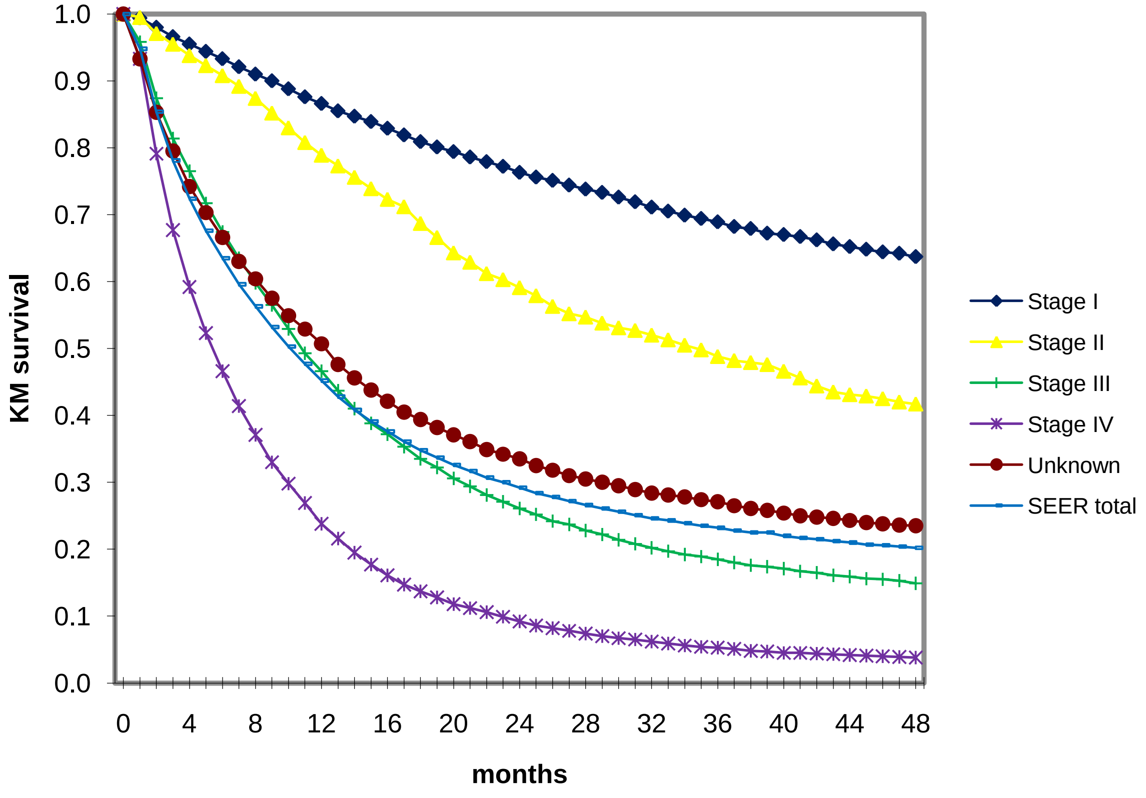

Table S2 Stage distribution of lung cancer observed in SEER 17 for the year 2000

Stage	Proportion
I	16.22%
II	2.91%
III	32.64%
IV	48.23%

The resulting KM estimate of the median survival time is 17 months between lung cancer diagnosis and death in the MDA patients as seen in the overall KM survival curve (Fig. S2). Figure S2 also shows SEER lung cancer specific survival curves for patients diagnosed during the year 2000. Details about the estimated healthy volunteer interval calculations are included in Table 3.

Table S3 Survival time from clinical diagnosis to death from lung cancer

Stage	MDA	SEER
I	66	54
II	41	27
III	18	10
IV	10	5
Unkown	N/A	10
Overall	17	11
Exponential mean (months)	24.5	15.9
Exponential mean (years)	**2.0**	**1.3**

SEER median survival time for LC-specific mortality is reported as 11 months [7]. The corresponding exponential distribution means are 2.0 years (MDA) and 1.3 years (SEER), and both are used to predict LC deaths in CARET. The following figure shows that the exponential distribution provides a reasonable approximation to the overall KM survival curve.

Figure S4 Comparison of Exponential Approximation and Overall KM Survival

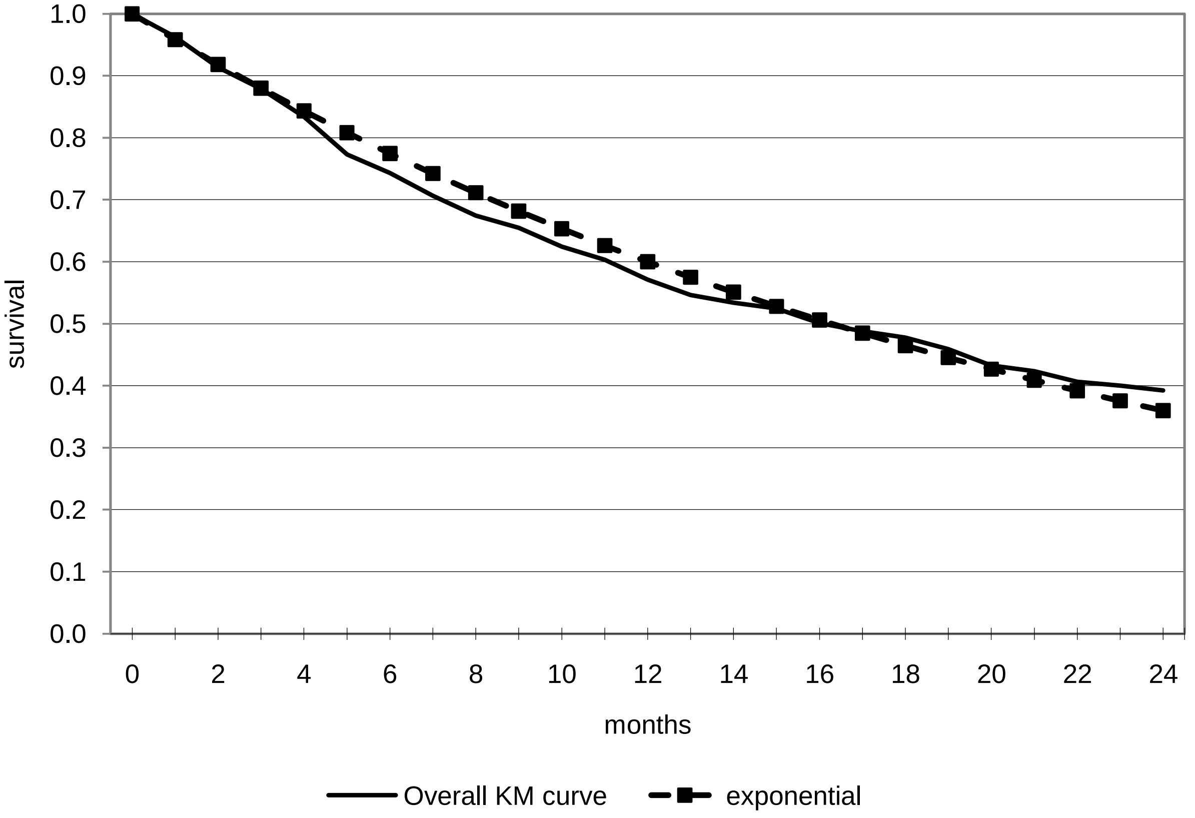

Derivation of the acceptance probability for eligibility-related bias adjustment Let us denote by X the age symptoms and by Y the time from symptoms to death. Let us assume X and Y are independent. Let us denote by A the age at enrollment. We are seeking expression for the acceptance probability, which can be defined as $\Pr\,[X > A|X + Y = t]$. Proceeding by definition of conditional probability, we obtain under the hypothesis that distribution of X has density $f_X(x)$ and that Y is distributed exponentially with parameter λ

$$\Pr[X > A|X + Y = t] = \lim_{\Delta t \to 0} \Pr[X > A|X + Y \in (t, t + \Delta t)]$$

where

$$\Pr[X > A|X + Y \in (t, t + \Delta t)] \cong \frac{\int_A^t f_X(u) \Delta t \lambda \exp\big(-\lambda(t-u)\big)\, du}{s(t)\Delta t}$$

$$= 1 - [s(A)/s(t)] \exp\,(-\lambda(t - A))$$

and $s(t)$ is the density of the distribution of $X + Y$. If we use our intuitive rejection strategy, we obtain $1 - \exp\,(-\lambda(t - A))$ instead. The following histogram shows the simulation distribution of the ratio of the theoretical and approximated acceptance probabilities (sim.prob, and theo.prob respectively in summary statistics below)

$$(1 - \exp\,(-\lambda(exit\ age - A)))/(1 - [s(A)/s(exit\ age)] \exp\,(-\lambda(exit\ age - A)),$$

which shows that the correction introduced by employing the exact expression is small.

```
summary(sim.prob)
   Min. 1st Qu.  Median    Mean 3rd Qu.    Max.
 0.6988  0.9950  0.9979  0.9868  0.9988  0.9999

summary(theo.prob)
   Min. 1st Qu.  Median    Mean 3rd Qu.    Max.
 0.7520  0.9951  0.9987  0.9886  0.9994  0.9999

ratio=sim.prob/theo.prob

summary(ratio)
```

Min.	1st Qu.	Median	Mean	3rd Qu.	Max.
0.9155	0.9979	0.9991	0.9980	1.0000	1.2180

Figure S5 Histogram of the ratio between simulated and derived acceptance probabilities for the individuals in CARET, both based on exponential survival time from diagnosis.

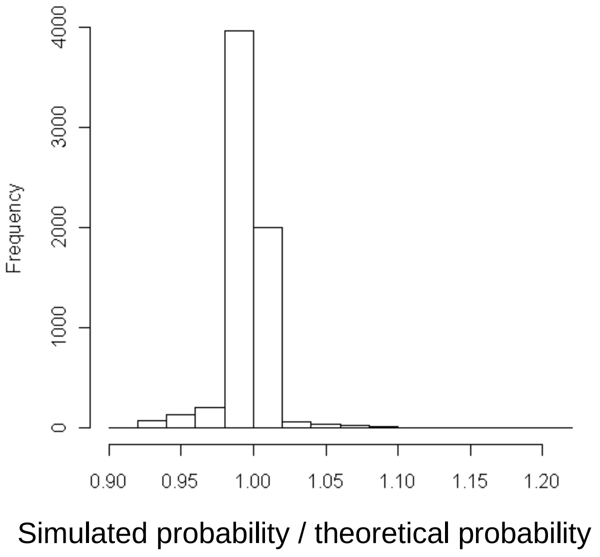

Further relaxing assumptions, we obtain under the hypothesis that distribution of X has density $f_X(x)$ and that Y is distributed with density $f_Y(y)$

$$\Pr[X > A|X + Y = t] = \frac{\int_A^t f_X(u)f_Y(t-u)du}{s(t)}$$

and $f_Y(y)$ is approximated by a Kaplan-Meier estimate as in Figure S4.

Figure S6 Histogram of the ratio between derived acceptance probabilities for the individuals in CARET, based on exponential survival time from diagnosis versus those based on KM estimate of the survival time from diagnosis.

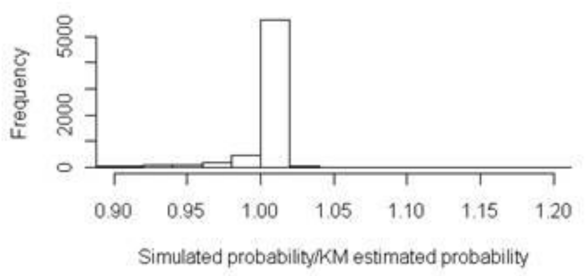

Exponential / KM estimate

Figures of person-years of follow-up and observed LC deaths by gender Comparison of person-years of follow-up and observed LC deaths in CARET give some indication that the healthy volunteer effect lasts through the first 3 years of follow-up and maybe last longer for men than women.

Figure S6 Observed LC deaths per follow-up year by gender

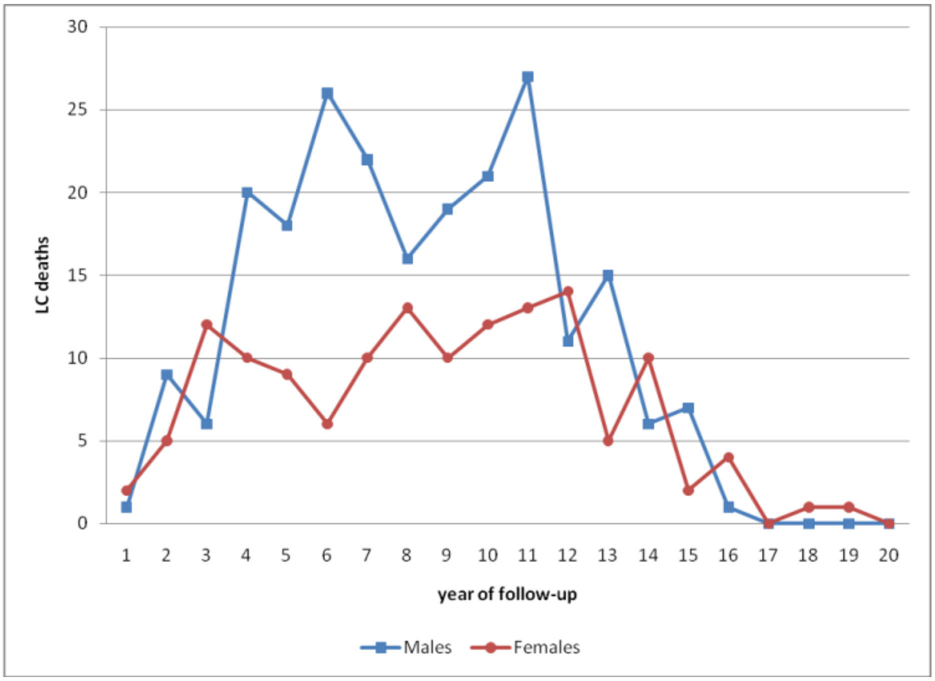

REFERENCES

1. Moolgavkar SH, Venzon DJ: **Two-event models for carcinogenesis: Incidence curves for childhood and adult tumours.** *Math Biosci* 1979, **47**: 55-77
2. Foy M, Spitz MR, Kimmel M, Gorlova OY: **A smoking based carcinogenesis model for lung cancer risk prediction.** (Submitted to International Journal of Cancer).
3. Heidenreich WF, Luebeck EG, Moolgavkar SH: **Some properties of the two-mutation clonal expansion model.** *Risk Anal* 1997, **17**(3): 391-399
4. Spitz MR, Wu X, Wang Y, Wang LE, Shete S, Amos CI, Guo Z, Lei L, Mohnrenweiser H, Wei Q: **Modulation of nucleotide excision repair capacity by XPD polymorphisms in lung cancer patients***.* *Cancer Res* 2001, **61**(4):1354-7
5. Thun MJ, Myers DG, Day-Lally C, Myers D, Calle EE, Flanders WD, Zhu B, Namboodiri MM, Heath Jr. CW: **Trends in tobacco smoking and mortality from cigarette use in Cancer Prevention Studies I (1959 through 1965) and II (1982 through 1988).** In: National Cancer Institute, *Smoking and Tobacco control, monograph 8: Changes in cigarette-related disease risks and their implication for prevention and control*. 1997
6. Meza R, Hazelton WD, Colditz GA, Moolgavkar SH: **Analysis of lung cancer incidence in the nurses' health and health professionals' follow-up studies using a multistage carcinogenesis model***.* *Cancer Causes Control* 2008, **19**(3):317-28
7. Surveillance Epidemiology, and End Results (SEER) Program (www.seer.cancer.gov) SEER*Stat Database: SEER 17 Regs Limited-Use + Hurricane Katrina Impacted Louisiana Cases, Nov 2008 Sub (1973-2006 varying) – Linked to County Attributes – Total U.S., 1969-2006 Counties, National Cancer Institute, DCCPS, Surveillance Research Program, Cancer Statistics Branch, released April 2009, based on November 2008 submission.
